# Transcriptome profiling of natural dichromatism in the annual fishes *Nothobranchius furzeri* and *Nothobranchius kadleci*

**DOI:** 10.1186/1471-2164-15-754

**Published:** 2014-09-02

**Authors:** Enoch Ng’oma, Marco Groth, Roberto Ripa, Matthias Platzer, Alessandro Cellerino

**Affiliations:** Leibniz Institute for Age Research – Fritz Lipmann Institute, Beutenbergstraße 11, 07745 Jena, Germany; Neurobiology Laboratory, Scuola Normale Superiore, Pisa, Italy

**Keywords:** Tail pigmentation, Functional Annotation Clustering, Melanin biosynthesis pathway, Xanthophore, Erythrophore, Muscle genes

## Abstract

**Background:**

The annual fish *Nothobranchius furzeri* is characterized by a natural dichromatism with yellow-tailed and red-tailed male individuals. These differences are due to different distributions of xanthophores and erythrophores in the two morphs. Previous crossing studies have showed that dichromatism in *N. furzeri* is inherited as a simple Mendelian trait with the yellow morph dominant over the red morph. The causative genetic variation was mapped by linkage analysis in a chromosome region containing the *Mc1r* locus. However, subsequent mapping showed that *Mc1r* is most likely not responsible for the color difference in *N. furzeri*. To gain further insight into the molecular basis of this phenotype, we performed RNA-seq on F2 progeny of a cross between *N. furzeri* male and *N. kadleci* female.

**Results:**

We identified 210 differentially-expressed genes between yellow and red fin samples. Functional annotation analysis revealed that genes with higher transcript levels in the yellow morph are enriched for the melanin synthesis pathway indicating that xanthophores are more similar to melanophores than are the erythrophores. Genes with higher expression levels in red-tails included xanthine dehydrogenase (*Xdh*), coding for a biosynthetic enzyme in the pteridine synthesis pathway, and genes related to muscle contraction. Comparison of DEGs obtained in this study with genes associated with pigmentation in the Midas cichlid (*A. citrinellus*) reveal similarities like involvement of the melanin biosynthesis pathway, the genes *Ptgir*, *Rasef* (RAS and EF-hand domain containing), as well as genes primarily expressed in muscle such as *Ttn* and *Ttnb* (titin, titin b).

**Conclusions:**

Regulation of genes in the melanin synthetic pathway is an expected finding and shows that *N. furzeri* is a genetically-tractable species for studying the genetic basis of natural phenotypic variations. The current list of differentially-expressed genes can be compared with the results of fine-mapping, to reveal the genetic architecture of this natural phenotype. However, an evolutionarily-conserved role of muscle-related genes in tail fin pigmentation is novel finding and interesting perspective for the future.

**Electronic supplementary material:**

The online version of this article (doi:10.1186/1471-2164-15-754) contains supplementary material, which is available to authorized users.

## Background

Pigmentation is a tractable phenotypic trait that can be easily quantified, and shows remarkable inter- and intra-specific variation. Differences in pigmentation are the most apparent trait that often distinguishes natural populations of a species and/or closely-related species [1–4]. Pigmentation has received considerable attention both in the context of speciation and adaptation [5–10]. Therefore, pigmentation represents a manageable case for studying molecular mechanisms underlying evolutionary processes. Much effort was invested in recent years to identify genetic pathways that determine pigmentation variations in humans and in natural animal populations that revealed a substantial conservation in the pathways controlling pigmentation in vertebrates [2, 8, 11, 12], with several genes associated with variations in mammal pigmentation that share the same function in teleost fish, such as *Kit*, *Mc1r*, *Oca2*, *Scl24a*, *Sox10*, *Mitf*, and *Ednrb* [8, 11].

Teleost fish are characterized by the existence of further pigmentation cells in addition to melanocytes (melanophores in fish). These are xanthophores (ochre or yellow), erythrophores (red), leucophores (whitish), iridophores (metallic or iridescent) and cyanophores (blue) [13]. Orange to red pigmentation can originate from carotenoids and broadly discussed as a marker of phenotypic quality or dietary history or endogenous pteridines [14–16]. A very common color variant in captive ornamental teleost species is the so-called "gold" or "orange" variant in which the melanophore density is reduced and the xanthophores become dominant giving the animals a yellow-orange color. A similar natural pattern is observed in Lake Malawi cichlids and is controlled by regulatory elements in the *Pax7* locus [4]. In Midas cichlids, pigmentation may change in the course of ontogeny giving rise to the gold morph, and a recent transcriptomic study identified genes regulated during this transition [2].

**Figure 1:**
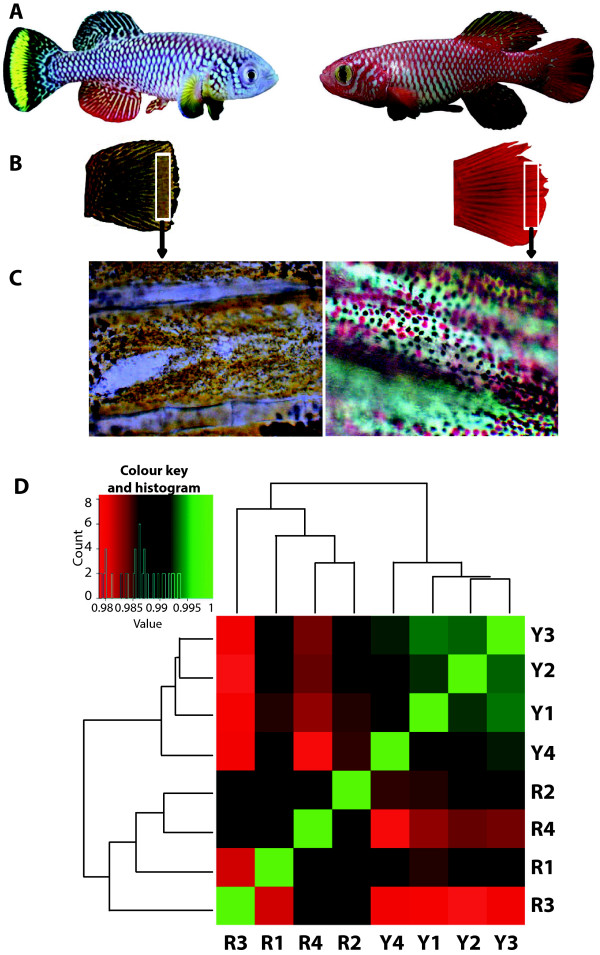
**Tail dichromatism in Nothobranchius fish.** **A**: The yellow morph of *N. furzeri* has a spotted tail with a sub-marginal yellow band and a marginal black band [Photo by Alexander Dorn]. *N. kadleci* have a solid red tail in general and sometimes with bluish spots in the proximal region. **B**: Corresponding yellow and red tail portions were clipped for RNA isolation. **C**: The yellow band of *N. furzeri* contains the yellow xanthophores almost exclusively, whereas the corresponding portions in *N. kadleci* have the black melanophores and red erythrophores (magnification, 10x). **D**: Hierarchical clustering of expression profiles derived from four RNA-seq pools of yellow (Y1 – Y4) and four pools of red (R1 –R4) tail samples. The profiles of samples correspond with the yellow and red tail phenotypic categories.

The African teleost *Nothobranchius furzeri* is the shortest-lived vertebrate that can be cultured in captivity and was recently introduced as a model organism in the context of aging research. A reference transcriptome for *N. furzeri* was recently generated [17]. *N. furzeri* is characterized by a striking dichromatism with two phenotypes: a "yellow-tail" morph that shows a yellow submarginal band and a black marginal band (Figure 1A), and a "red-tail" morph with a broad red marginal band. These two morphs coexist over a broad portion of the distribution range in Southern Mozambique, with the yellow morph predominating in the margin of the distribution range [18]. The two morphs are not genetically differentiated implying the absence of assortative mating [19, 20]. The sister taxon to *N. furzeri* is *N. kadleci* [20], with parapatric distribution and showing a solid red tail. Previous crossing studies have revealed that dichromatism in *N. furzeri* is inherited as a simple Mendelian trait with the yellow morph dominant over the red morph [21]. The causative genetic variation was mapped by linkage analysis in a chromosome region containing the *Mc1r* locus*,* but subsequent mapping of an *Mc1r* marker showed that the gene is most likely not responsible for the color difference in *N. furzeri* [20, 21].

In the present paper, we used RNA-seq to identify genes differentially expressed in the yellow vs. red morphs of *N. furzeri* and *N. kadleci.*

## Results and discussion

### Tail expression profiles correspond with dichromatism

To make sure that genetic mechanisms of tail pigmentation are identical in the two species, we performed a complementation test by crossing individuals of the MZM-0703 red-tailed strain of *N. furzeri* [22] with those of *N. kadleci* in both possible combinations of parents. All 24 F1 male offspring from the two crosses showed a red tail supporting the assumption that the same *locus* is associated with red colour in both species.

In order to identify differences in gene expression between the two colour morphs that are independent of the genetic background, we used a F2 hybrid panel obtained by crossing a yellow-tailed male of the *N. furzeri* strain GRZ with a female *N. kadleci*. A clear segregation of the two phenotypes was observed in F2 generation (Ng’oma, unpublished data) confirming the Mendelian inheritance of this trait [21]. At the age of 16 weeks, the yellow submarginal band of the caudal fin and the corresponding region of red-tailed fish were excised taking care of excluding the black marginal band (Figure 1B,C). Four RNA pools of each colour morph (each pool containing samples of four fish) were analyzed by Illumina RNA-seq resulting in 334,588,906 reads (163,622,787 for red sample pools and 170,966,119 for yellow sample pools) having an average of around 42 million reads per sample pool. About 55% of the reads could be mapped to the reference transcriptome of *N. furzeri*. Hierarchical clustering of the reads showed that the samples group in correspondence with the yellow and red phenotypic categories (Figure 1D). In total, 210 differentially-expressed genes (DEGs) were identified by the intersection of the statistical tests in the packages edgeR and DEseq (see also Methods). Of the 210 DEGs, 119 were up regulated, while 91 were down-regulated in the yellow samples with respect to the red samples. A full list of DEGs is provided in Additional file 1. Project data are deposited in the Short Reads Archive (SRA), reference number SRP034010.

**Figure 2:**
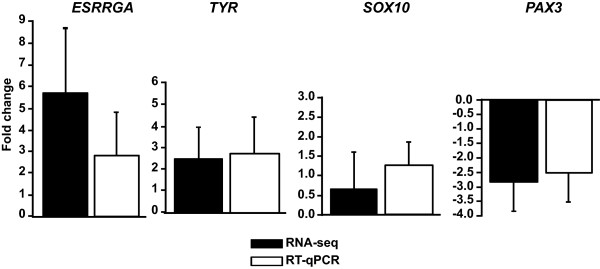
**RT-qPCR validation of sample genes regulated in RNA-seq data.** Four sample genes were selected based on fold change or known role in melanocyte biology (log_2_ fold change yellow vs. red morph). The same individuals whose expression was quantified with RNA-seq were used in qPCR. Expression was normalized to *TBP*, as internal control; error bars represent standard deviation.

In order to confirm the expression changes detected by RNA-seq we performed qPCR for four sample genes selected on the basis of fold change (log_2_ fold change yellow vs. red morph; *P* value of adjusted meanof DESeq and edgeR) or known role in pigmentation biology: *Esrrga* (5.66; *P* = 6.74×10^-19^), *Tyr* (2.51; *P* = 3:29×10^-08^), *Pax3* (-2.82; *P* = 1.75×10^-16^), and *Sox10* (0.67; *P* = 5.29×10^-3^). Estrogen-related receptor gamma a (*Esrrga*) was the gene with highest fold change in yellow samples, Tyrosinase (*Tyr*) is a rate-limiting biosynthetic enzyme of melanin, and the transcription factors *Pax3* and *Sox10* are important in the differentiation of melanocytes [11, 23]. In all four cases, the direction of change was consistent between the two methods (Figure 2). Thus, as already shown [24], we found coherence between RNA-seq and qPCR.

### Melanogenesis is the main process regulated between morphs

**Figure 3:**
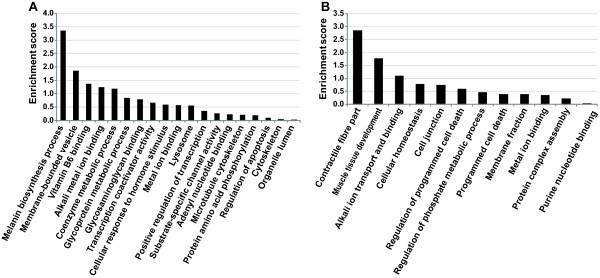
**Gene Ontology.** DAVID Functional Annotation Clustering (FAC) analysis of differentially expressed genes (DEGs) obtained by comparison of yellow vs. red tail RNA samples. **A**. Enriched functional gene clusters for the 119 up-regulated genes. **B**. Enriched functional gene clusters for the 91 down-regulated genes. Significance is determined by corresponding enrichment scores.

To assess which biological processes may be modulated by the DEGs, Gene Ontology (GO) analysis was performed. Lists of genes with significantly different transcript levels were analyzed with the Functional Annotation Clustering (FAC) tool implemented in the Database for Annotation, Visualization and Integrated Discovery (DAVID) [25]. FAC allows clustering of GO categories sharing significant amount of genes and partially obviates to redundancy of GO classes by providing a more compact output than conventional GO enrichment analysis. The FAC analysis of 119 higher expressed genes resulted into 19 enriched functional clusters under the high stringency option. FAC1 (pigmentation and melanogenesis) was the most biologically important gene group with enrichment score of 3.54 (Figure 3A). Melanin biosynthetic process (GO: 0042438) was the most significant biological process (*P* = 7.9E×10^-06^) in yellow tails (see Additional file 2). FAC analysis of the 91 lower expressed genes yielded 12 enriched functional gene groups. Muscle components and organization were the most significant biological processes, such as Z disc (*P* 0 7.6E^-04^), myofibril (*P* = 9.1E^-04^), and contractile fibre part (*P* = 9.8E^-04^) (Figure 3B; Additional file 2).

FAC1 revealed five pigmentation genes (*Dct*, *Tyrp1*, *Tyr*, *Oca2*, and *Sox10*) with fold-change range of 1.8 – 7.9 (Additional file 2). *Tyr*, *Tyrp1* and *Dct* are members of the tyrosinase gene family that arose by ancient duplications and catalyse conversion of amino acid tyrosine to melanin[26]. Deletions in *Oca2* were shown to be responsible for parallel evolution of albinism in the Mexican cave fish [10]. *Sox10* in together with *Mitf* regulate melanocyte differentiation by activating *Tyr* [27, 28]. The remaining FAC clusters of up-regulated genes were dominated by solute carrier and channel genes including *Slc24a5*, presumably a cation exchanger linked with variations in number, size and density of melanosomes in humans and zebrafish [29]. Muscle-related processes featured prominently in the FAC clusters with lower transcript levels (Additional file 2). Cluster 3 included *Xdh* (xanthine dehydrogenase), 2.1-fold lower expressed which is a key enzyme in the synthesis of pteridine pigments. This gene group also contained solute carrier protein genes including *Ano8* (anoctamin 8). Under relaxed clustering stringency, *Mreg* (melanoregulin) was also observed.

The enrichment of melanogenesis-related terms with respect to higher expression in yellow fish suggests that melanocytes are found more often in yellow- with respect to red-tailed fish. However, tail micrographs (Figure 1C) surprisingly showed that the yellow submarginal band actually lacked melanocytes, which were abundant in corresponding red tail regions. This suggests that the xanthophores were connected with melanogenesis, thus indicating that xanthophores are functionally more related to the melanocytes and most likely ontogenetically closer to the melanocyte lineage than to the erythrophores. Indeed, studies have reported on the role of *Pax3*/*Pax7* fate switch to myogenesis [30] in which pro-myogenesis Hedgehog and Fgf8 signalling suppress *Pax3*/*Pax7*, and promote myogenic regulatory factors. A further study on the role of *Pax3* and *Pax7* genes in the zebrafish neural crest [31] demonstrated sequential actions of *Pax3* and *Pax7* in fate switch induction from xanthophores to melanophores in *Pax3* knock-down embryos. Higher expression of *Xdh* in the red-tail samples indicates that the red pigment is pteridine-based. Both sepiapterin and drosopterin are formed from 6-pyruvoyl-H4pterin, and *Xdh* is required for the formation of yellow pigments from sepiapterin [11]. However, the pathway leading to the formation of the orange-red pigments from drosopterin is yet to be elucidated. The regulation of *Xdh* in this study may suggest a possible role of this gene in Nothobranchius tail pigmentation.

### Regulation of muscle genes in Midas cichlids

Regulation of genes in the melanin synthetic pathway is an expected finding. A surprising observation is the regulation of muscle-related genes. The tail is a heterogeneous source of RNA, containing various other cells (bone, cartilage, etc.) in addition to pigment cells. We compared observed differences between Nothobranchius colour morphs with expression changes observed in the Midas cichlids in relation to transition from the grey to the gold morph [2] and identified 9 genes with consistent expression changes in the two species (Table 1). It is interesting that just a handful of muscle genes were regulated, and that muscle genes were regulated in both Nothobranchius and Midas.

**Table 1:** **Genes that were differentially expressed with respect to dichromatism in both Nothobranchius and Midas cichlids**

Gene symbol	Expression (log _2_ foldchange)	Gene official name
TYRP1	2.98	tyrosinase-related protein 1
TYR	2.50	tyrosinase (oculocutaneous albinism IA)
PMEL	2.33	premelanosome protein
SLC6A15	1.84	solute carrier family 6 (neutral amino acid transporter), member 15
PTGIR	1.11	prostaglandin I2 (prostacyclin) receptor (IP)
RASEF	-1.06	RAS and EF-hand domain containing
MREG	-1.13	melanoregulin
TTNB	Inf.*	titin b
TTN	Inf.*	titin

*no reads in yellow RNA samples.

We noted above the role of known colour genes (*Pax3*/*Pax7*) in muscle development. *Pax3* plays key roles in melanocyte development (proliferation, survival and migration), and melanoma by maintaining a less differentiated ‘stem’ cell-like phenotype [32]. Further, a *cis*-regulatory mutation in *Pax7* drives the differentiation of the orange-blotch phenotype from the common male nuptial colouration in Lake Malawi cichlids [4]. Similarly, the intensity of coat colour is reduced in mice lacking myosine heavy chain genes such as *Myo5a* (or *Myha*) due to loss of connections between melanosomes and F-actin which affect melanosome distribution in the cell [33, 34]. It would be worthwhile to analyze gene expression at the embryonic stage when cell fate specification and differentiation is still underway. The challenge though would be in distinguishing embryo colour morphs given that the manner and timing in which erythrophores and xanthophores differentiate from their precursor is not understood. Taken together, these observations and our results suggest a possible link between the pigmentation and mayogenic pathways in Nothobranchius. Presently, the functional significance of these genes for red pigmentation remains unknown and forms an interesting perspective for the future.

## Conclusions

This study successfully identified pigmentation as the major biological process altered between tail colour morphs consistent with knowledge in other species. Our results therefore show that *N. furzeri* is a genetically-tractable species for studying the genetic basis of natural phenotypic variations, not only through classical linkage methods as in [21], but also amenable to modern hi-tech genomic approaches. Thus, the current list of differentially-expressed genes can feed back to, and be compared with the results of fine-mapping of the colour locus that is ongoing, to reveal the genetic architecture of this natural phenotype.

## Methods

### Fish breeding and sample preparation

A male of the yellow tail morph, *N. furzeri* (GRZ strain) was mated to a female of the red tail morph, *N. kadleci* resulting into 34 F1 fish. From sib mating of several F1 pairs, 16 yellow tailed and 16 red tailed F2 fish were sacrificed at 16 weeks of age for transcriptome comparison. Care and experimentation with fish followed protocols approved by the local authority in the State of Thüringia (Veterinaer- und Lebensmittelueberwachungsamt). Yellow sub-marginal strips of the tailfin and corresponding regions of red-tailed fish were excised (Figure 1B) and transferred to 1.5 ml tubes containing 600 μl RNAlater (Qiagen, Hilden, Germany) and stored at -20°C until RNA isolation.

### RNA sequencing

Total RNA was isolated using QIAzol (Qiagen) following manufacturer’s protocol with modifications as described elsewhere [35]. Four pools, each containing 1 μg RNA, were prepared for each phenotype by combining RNA from four fish in equimolar amounts. RNA samples were checked for quality using an Agilent Bioanalyzer 2100 in combination with the Agilent RNA 6000 Nano Kit (Agilent Technologies) to have a RIN value > 7. Libraries were prepared using Illumina’s TruSeq™ RNA Sample Prep Kit v2 (Illumina Inc.) following the manufacturer’s description, and sequenced with Illumina HiSeq 2000 in 50 bp single-read mode.

### Identification of differentially expressed genes

The resulting reads were mapped to nonredundant set of transcript contigs representing the longest transcript contig per gene of *N. furzeri* [17] using ELAND (Illumina Inc.). Based on the mapping results the reads per transcript were counted. The count data were used for determination of differentially expressed genes (DEGs) using the packages edgeR [36] and DESeq [37] within the statistical environment R (R Development Core Team, 2012). The p-values were adjusted for multiple testing using the Benjamini-Hochberg algorithm [38]. Genes were regarded to be differentially expressed when both tests showed a significance ≤ 0.01. This resulted in a total number of 210 genes which were found to be differentially expressed.

### Gene ontology analysis

Hierarchical clustering was performed to sort samples and genes with similar profiles using a dedicated R-script, count_genes_per_transcript.pl [39]. Gene Ontology analysis to identify biological processes likely modulated by the DEGs was performed in DAVID tools [40]. All analysis in DAVID was performed using the Functional Annotation Clustering (FAC) module set to high stringency. The FAC enrichment score (-log_10_P-values/n) for each cluster was graphed. The enrichment score gives indication of the biological significance of the clusters [41]. Tail micrographs were captured from corresponding yellow and red sub-marginal regions at 10× using the AxioVision Product Suite (Carl Zeiss, Jena, Germany).

### Validation by RT-qPCR

cDNA was synthesized in 20 μl volumes for each sequenced individual’s RNA using the QuantiTect® Reverse Transcription Kit (Qiagen) following the supplier’s protocol, and diluted with 200 μl ultrapure water. PCR primers for a sample of genes regulated in RNA-seq including *Esrrga*, *Tyr*, *Sox10*, and *Pax3* were designed from CDS across introns (forward and reverse): *Esrrga* - GAGGATAGGGAAGAGAAG and AACAGAGAGCAGTGGACG; *Tyr*- GCTCTGTCTTCTCTTCTTG and ATGTTGGCGGTGCGGTCC; *Sox10* - ATCAGACGACGAAGAGGAG and GCAGGTGGGGGTGTTGG; *Pax3* - GGGAAGGAGGCTGGATAG and CGTGGGTAGTTCTGGTGAG. The tatabox binding protein (*Tbp*– CGGTTGGAGGGTTTAGTCCT and GCAAGACGATTCTGGGTTTG) was used for normalization. Real-time PCR was performed with the RotorGene 6000 (Qiagen) GoTaq® qPCR Master Mix (Promega) using 4 ng cDNA per reaction in triplicate on the following temperature profile: 95°C for 2 min; 35× (95°C, 30 s, 60°C, 30 s, 72°C, 25 s). Statistical analysis of RT-qPCR results was conducted with the relative expression software tool, REST (Qiagen) [42], which implements a mathematical model comparing treatment and control samples and significance is tested by a randomization test.

## Electronic supplementary material

Additional file 1: **CSV: List of 210 differentially expressed genes identified in the intersection of DESeq and edgeR.** (CSV 27 KB)

Additional file 2: **CSV: DAVID Functional Annotation Clustering results of up-regulated (A) and down-regulated (B) gene lists.** Only gene groups containing significant GO terms are shown. The genes are listed below each cluster. (CSV 4 KB)
